# Deep Machine Learning Techniques for the Detection and Classification of Sperm Whale Bioacoustics

**DOI:** 10.1038/s41598-019-48909-4

**Published:** 2019-08-29

**Authors:** Peter C. Bermant, Michael M. Bronstein, Robert J. Wood, Shane Gero, David F. Gruber

**Affiliations:** 1 Radcliffe Institute for Advanced Study, Harvard University, Cambridge, MA USA; 2 Department of Computing, Imperial College, London, MA UK; 3 Wyss Institute for Biologically Inspired Engineering, Harvard University, Cambridge, MA USA; 4 Harvard John A. Paulson School of Engineering and Applied Sciences, Harvard University, Cambridge, MA USA; 5 Department of Zoophysiology, Institute for Bioscience, Aarhus University, C.F., Møllers Allé 3, Aarhus, 8000 Denmark; 6 Department of Natural Sciences, Baruch College and The Graduate Center, PhD Program in Biology, City University of New York, New York, NY USA; 7 Twitter, 20 Air Street, London W1B 5DL, United Kingdom

**Keywords:** Computational biology and bioinformatics, Zoology, Mathematics and computing

## Abstract

We implemented Machine Learning (ML) techniques to advance the study of sperm whale (*Physeter macrocephalus*) bioacoustics. This entailed employing Convolutional Neural Networks (CNNs) to construct an echolocation click detector designed to classify spectrograms generated from sperm whale acoustic data according to the presence or absence of a click. The click detector achieved 99.5% accuracy in classifying 650 spectrograms. The successful application of CNNs to clicks reveals the potential of future studies to train CNN-based architectures to extract finer-scale details from cetacean spectrograms. Long short-term memory and gated recurrent unit recurrent neural networks were trained to perform classification tasks, including (1) “coda type classification” where we obtained 97.5% accuracy in categorizing 23 coda types from a Dominica dataset containing 8,719 codas and 93.6% accuracy in categorizing 43 coda types from an Eastern Tropical Pacific (ETP) dataset with 16,995 codas; (2) “vocal clan classification” where we obtained 95.3% accuracy for two clan classes from Dominica and 93.1% for four ETP clan types; and (3) “individual whale identification” where we obtained 99.4% accuracy using two Dominica sperm whales. These results demonstrate the feasibility of applying ML to sperm whale bioacoustics and establish the validity of constructing neural networks to learn meaningful representations of whale vocalizations.

## Introduction

While human language epitomizes a peak in communicative complexity across biological taxa, cetaceans represent an important taxon for testing hypotheses relating to the evolution and development of sophisticated communication systems. Despite the absolute magnitude and highly-developed neuroanatomical structure of the cetacean brain, the extent to which cetaceans possess a grammatical-syntactical language remains uncertain^1^. Regardless, cetaceans have cognitive abilities^2^ and societies^3^ that are commensurate with those of human phylogenetic relatives^4^, but their ocean habitat provides a difference in ecology, which can be revealing from a comparative perspective, especially with regards to evolutionary adaptations required for aquatic communication^5^. In contrast to the relative ease of investigating the communicative capabilities of humans and other terrestrial organisms, working with non-captive animals in the ocean environment presents numerous technical and logistical difficulties^6^. Given these challenges, the development of improved computational techniques for analyzing cetacean sounds plays an important role in enabling researchers to address questions pertaining to cetacean vocal behavior. In particular, automated methods are increasingly employed to detect, classify, and identify cetacean sounds and to efficiently process acoustic data without human bias^7^, which has significantly expedited and advanced the study of cetacean bioacoustics.

Modern analysis of human speech and language often takes advantage of Machine Learning (ML) techniques, which entail the design and development of self-learning algorithms that allow computers to evolve behaviors based on empirical data, such as from sensors and databases. In general, ML problems can be divided into three major subcategories: supervised learning, unsupervised learning, and reinforcement learning. Unsupervised learning is concerned with uncovering structure within a dataset and extracting meaningful information, oftentimes without prior knowledge of how the data are organized^8^. Supervised learning relies on prior knowledge regarding an example dataset in order to make predictions about new, unseen data points (“generalization”). This is accomplished through the use of a training set comprised of labeled data, whose ground truth response values are known, in order to train the predictor. Since supervised learning typically requires abundant labeled data, various techniques can be employed to alleviate this requirement, including pretraining on some different (“proxy”) task for which labeled data are easier to obtain^9^. Reinforcement learning involves the development of software agents that interact with the environment so as to maximize a numerical reward signal^10^.

The predominant modern ML paradigm is Deep Learning (DL), a representation learning method in which the machine automatically discovers the representations that are required for carrying out a feature detection or classification task using raw input data. In particular, DL enables multilayer computational models to learn representations of data through the hierarchical composition of relatively simple non-linear modules that transform features into progressively higher levels of abstraction^11^. Effectively, by proceeding from low-level to high-level feature abstraction, deep networks—which can be constructed from many layers and many units within layers—are able to learn increasingly complex functions^12^. Importantly, the higher-level representations allow deep networks to extract relevant features from input data^11^, which can be used to accurately perform discrimination, classification, and detection tasks. Artificial Neural Networks (ANNs) are popular realizations of such deep multilayer hierarchies, acting as highly non-linear parametric mappings from the input to the output space, with parameters (“weights”) determined by the optimization of some objective function (“cost”). This optimization is performed by backpropagating the error through the layers of the network in order to produce a sequence of incremental updates of the neural network weights^13^. Popular among a plethora of different ANN architectures are Multi-Layer Perceptrons (MLPs) typically used for general classification and regression problems, Convolutional Neural Networks (CNNs) for image classification tasks, and Recurrent Neural Networks (RNNs) for time series sequence prediction problems.

In the past decade, growing computational powers and the enhanced availability of large collections of labeled data have enabled the successful construction and training of neural networks with many layers and degrees of freedom^11^, yielding innovative breakthroughs in a variety of tasks in speech recognition^9,14^, machine translation^15^, image analysis, and computer vision^16^. As of today, revolutionary algorithms in these fields are primarily based on DL techniques. Furthermore, DL has matured into a technology that is widely used in commercial applications, including Apple’s Siri speech recognition, Google’s text translation, and Mobileye computer vision-based technology for autonomously-driving cars^17^.

Cetacean bioacoustics has been an area of increasing study since the first recordings of marine mammals in the late 1940s^18^. Recently, ML-based methods have been applied to cetacean vocalizations, including the use of unsupervised self-organizing networks to categorize the bioacoustic repertoire of false killer whale (*Pseudorca crassidens*) vocalizations^19^. Gaussian Mixture Models (GMMs) and Support Vector Machine (SVM) algorithms have been used to construct cetacean species detectors to discriminate between signals produced by Blainville’s beaked whales (*Mesoplodon densirostris*), short-finned pilot whales (*Globicephala macrorhynchus*), and Risso’s dolphins (*Grampus griseus*)^20^; and similar computational techniques have been implemented in an effort to estimate sperm whale size distributions^21^. It has been demonstrated that a radial basis function network could effectively distinguish between six individual sperm whales^22^; and similarly, Hidden Markov Models (HMMs) and GMMs have performed the automatic identification of individual killer whales (*Orcinus orca*)^23^. In addition, ANNs have been constructed to classify the bioacoustic signals of killer whales based on call type, individual whale identity, and community dialect^24,25^. Most recently, Google obtained 90% accuracy using a ResNet-50 architecture, typically used for image classification, to develop a CNN-based humpback whale (*Megaptera novaeangliae*) detector for classifying non-speech audio^26^.

Among cetaceans, the sperm whale is known to have a sophisticated communication system^27–29^, based largely codas, which are stereotyped sequences of 3–40 broadband clicks that are, in general, transmitted between socializing whales^27,29^. The sperm whale communication system is thought to mediate a multileveled social structure^30^. Female and immature sperm whales live in small social units with stable, long-term membership often made up of kin^29,31–34^. In the Caribbean, units show decade-long social preferences to associate with specific other units into “Groups”^35^, while in the Pacific, no such preferences among units have been found^34^. All units which share a vocal dialect, made up of over 20 different coda types, are said to belong to the same vocal clan. Socially segregated, but sympatric, clans have been found in both the Pacific and Atlantic Oceans^29,36^. Clans appear to demonstrate differences in horizontal movement, social behavior, dive synchronies, diet, and foraging tactics, which may affect fitness of constituent units^37–42^. Sperm whale codas appear not only to encode clan identity, but they also may contain unit- and individual-level acoustic specificity^43–45^, which suggests that codas could potentially serve to identify individuals, social units, and the dialect as a whole. Differential coda production might function to delineate socially segregated, sympatric clans^44^. In addition to the communicative codas, sperm whales generate an extensive variety of click-based sounds, including (1) usual clicks for echolocation and foraging^46–48^, (2) buzzes for short-range prey detection and feeding^49–51^, and (3) reverberating slow clicks, also known as clangs, produced only by mature males^47^, in concert with other rare call types that have been poorly characterized. Given the complexity of its acoustic behavior, the sperm whale serves as an ideal species for constructing, applying, and testing novel computational methods to improve the analysis of cetacean bioacoustics.

However, the study of sperm whale communication has been slowed by the immense time investment required to collect high-quality audio recordings in the field and then subsequently to analyze and annotate these recordings manually prior to being able to answer novel questions regarding communicative function of signals or information exchanged between animals. Here, we use Neural Network (NN)-based ML techniques pioneered in the study of human speech and language^9,52^ and apply them to sperm whale vocalizations to accelerate the abilities of researchers to address questions about cetacean communication systems. Specifically, we undertake four primary tasks, including (1) detection of echolocation clicks using a CNN-based approach, (2) classification of codas into categorical types using a Long Short-Term Memory (LSTM) RNN-based method, (3) recognition of vocal clan coda dialects with LSTM RNNs, and (4) identification of individual whales based on coda production with LSTM RNNs. LSTM RNNs are effective for this purpose since their architecture enables the network to preserve the non-independent sequential order of the input data^8^. As an alternative to LSTM, we also report results with a Gated Recurrent Unit (GRU) architecture, a similar but simpler RNN with lower computational complexity and fewer parameters than LSTM (the latter being important in our setting of little data due to a lower risk of overfitting). This study serves as a first step towards the development of an end-to-end system capable of automated detection, classification, and prediction based on audio recordings of sperm whale sounds.

## Results

### Using a CNN-based approach to detect sperm whale echolocation clicks

**Figure 1:**
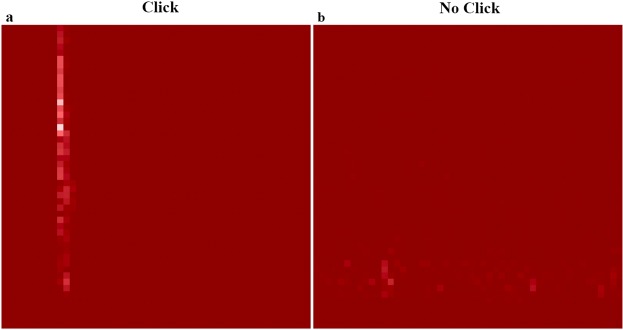
Input testing spectrogram images with the trained network’s predicted output labels of (**a**) Click and (**b**) No Click. The lack of labeled axes and the image resolution reflect that these are the images that are used purely as input data when training the CNN. The resolution suffices for training a CNN-based echolocation click detector.

We achieve a training detection accuracy of 99.5% using 650 spectrogram images, including 325 from each class (click vs. no click) (see Supplementary Fig. S1). We optimize the model by performing an exhaustive grid search through the high-dimensional hyperparameter space. Using 100 unseen images (50 belonging to each class) reserved for testing the trained network, the model categorizes all 100 images according to the correct annotation with zero mislabeled spectrograms (100% accuracy) (Fig. 1).

### Using LSTM and GRU RNN-based approach to classify sperm whale codas

One of the challenges of supervised learning is the need for labeled data that, in some cases, might be hard to obtain. An approach recently gaining popularity (e.g. in computer vision) is that of “self-supervised” learning (see^53^), wherein one defines a “proxy task” bearing some similarity to the main problem, in order to exploit large-scale data for which the labels are easier to obtain. The neural network trained this way often produces features relevant for the main task (“transfer learning”) and can be fine-tuned on a smaller dataset, optimizing the cost function associated with the main task. This procedure involves training a “base” network to carry out the proxy task and transferring the learned representations to a “target network” that is subsequently trained to perform the main task^54^.

We follow a similar philosophy here, initially pretraining a deep LSTM base model to perform a proxy task of time series prediction of the temporal distance between clicks *n* and *(n-1)* in an *n*-click coda, given the first *(n-1)* clicks in the coda (see Supplementary Fig. S2). Training this network involves minimizing the mean squared error (MSE) between the ground truth and predicted Inter-Click Interval (ICI) values (see Supplementary Fig. S3); the ground truth labels (ICI values) are abundantly available for this proxy task. Prior to training, the relative error of the prediction can exceed 300% while following training, the error can be reduced to as low as 12.4%. This suggests that this pretraining procedure enables the base network to extract meaningful features and representations from the time series coda sequence inputs. However, the task of predicting the temporal position of the last click in the coda is not necessarily of express interest in the study of sperm whale vocal behavior and is used as a proxy task only^55^.

After pretraining the model, we implement the transfer learning procedure, in which we save the layers of the pretrained network as fixed (untrainable) parameters and add shallow trainable layers, the weights of which are determined during subsequent training on the main tasks of coda type, vocal clan, and individual whale identity (ID) classification. The use of pretrained layers greatly simplifies the architecture, reduces the number of parameters, and makes it possible to train the model for the main task given the constraints associated with using a relatively small labeled dataset. With three annotated labels (coda type, vocal clan, individual whale ID) present in the dataset, we construct three different models, each optimized for the particular task. During the training phase, the target network adjusts the trainable parameters in such a way as to minimize the objective loss function, which, in this case, is the conventional categorical cross entropy function. As training progresses, accuracy (defined as the fraction of coda inputs labeled appropriately) increases (see Supplementary Fig. S4). Following training using the Caribbean dataset, the losses for the coda type, clan class, and whale ID networks are 0.018, 0.160, and 0.071, respectively, and the respective accuracies are 99.4%, 95.1%, and 98.3%. Evaluating the networks using 8,032 codas labeled by coda type, 1,898 codas labeled by clan class (949 belonging to each class “EC1” and “EC2”), and 516 codas labeled by whale identity (258 belonging to each whale #5722 and #5727), we obtain respective accuracies of 97.5%, 95.3%, and 99.4%. For the clan class and whale ID tasks, we ensure to normalize the datasets such that each of the two labels present comprise half of the total dataset as a means to remove potential bias. In an effort to address possible overfitting, we repeat the analysis, ensuring to segment the codas into separate training and testing datasets. For the coda type network, we train the model using 90% of the 8,032 codas and obtain an accuracy of 99.7%, and we test the model on 804 unseen codas, yielding an accuracy of 99.9%, suggesting that the trained model generalizes effectively to data. Carrying out this procedure for the clan classifier, we train the model on 1,708 codas (95.1% accuracy) and test on 190 unseen codas, achieving an accuracy of 96.8% using the two clans “EC1” and “EC2”. Given the relatively small number of codas labeled by whale ID, we opt to evaluate the model using the entire dataset. These results suggest the validity of employing ML-based techniques to advance the study of sperm whale bioacoustics and provide a practical technique to deal with the scarcity of labeled data by using self-supervised pretraining on a proxy task. In addition, the results of the clan class and whale ID analyses indicate clan-level and individual-level characteristics of sperm whale vocalizations, in accordance with the findings of^43,44^. Thus, not only do our results provide novel computational methods and techniques; they also offer important biological insight into sperm whale vocal behavior.

**Figure 2:**
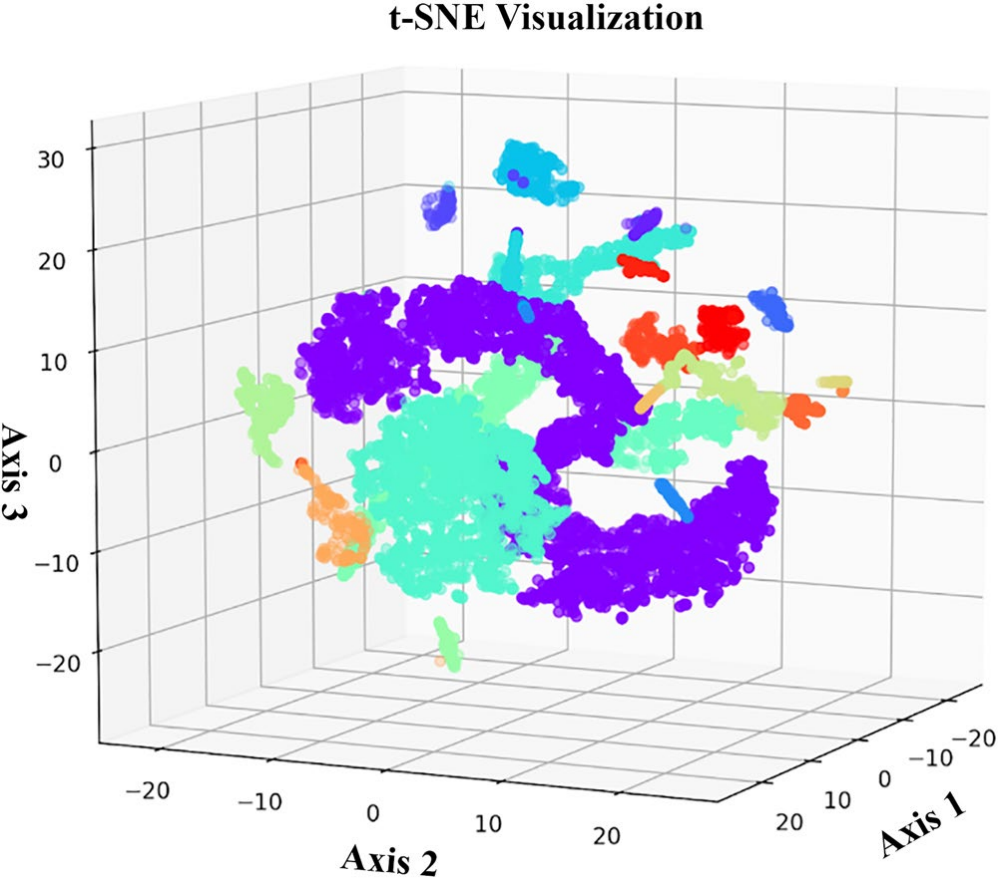
t-SNE visualization of the coda type classifier with colors denoting different coda type. Standard PCA and t-SNE techniques are used to plot the coda type hidden features in a three-dimensional feature space.

**Figure 3:**
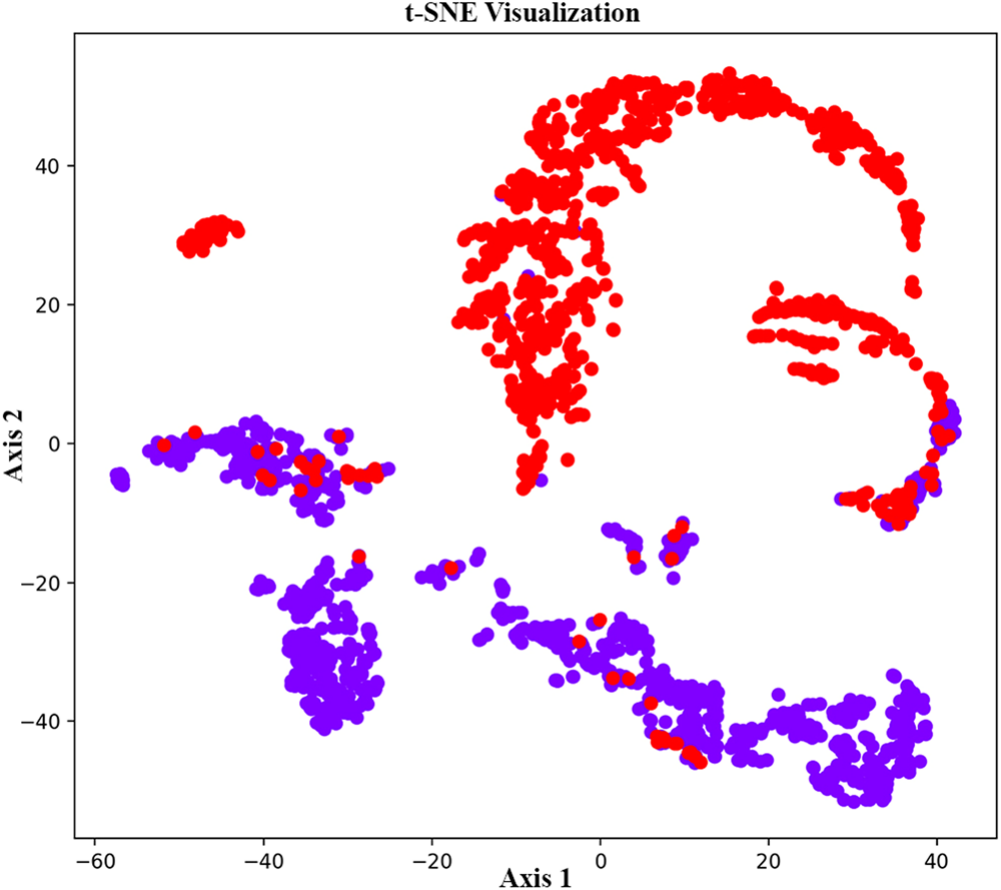
t-SNE visualization of the vocal clan classifier with the two classes representing the two clans identified in the eastern Caribbean (purple points “EC1”, red points “EC2”). We implement PCA and t-SNE to visualize the hidden features of the clan class network.

**Figure 4:**
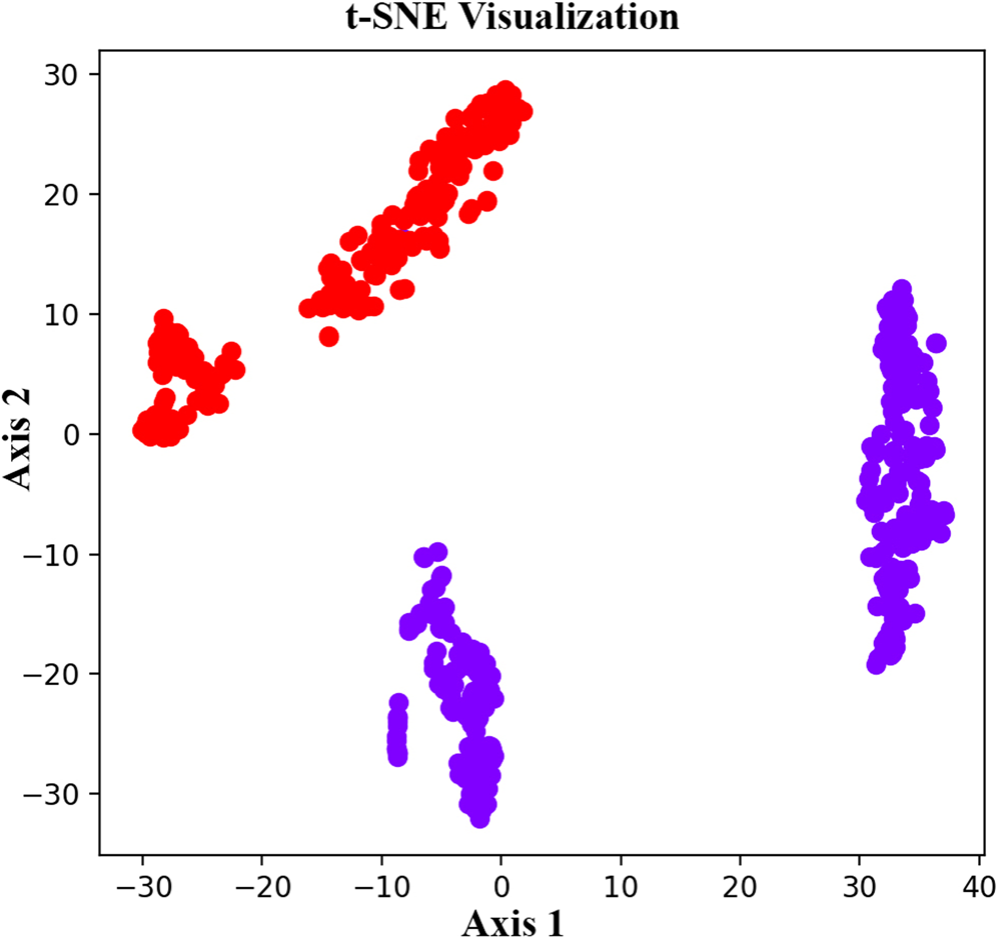
t-SNE visualization of the whale ID type classifier distinguishing between two identified whales from the “EC1” clan recorded off Dominica (purple points indicate codas produced by whale #5722, and the red points are codas generated by whale #5727). We implement PCA and t-SNE to visualize the hidden features in the whale ID classifier network.

To visualize the activations of the networks, we employ the t-Distributed Stochastic Neighbor Embedding (t-SNE) algorithm to reduce the dimensionality of the hidden features within the models and to plot the outputs of the models in lower-dimensional feature spaces. We use Principal Component Analysis (PCA) to reduce the dimensionality of the feature space from 256 to 20, followed by t-SNE to further reduce the dimensionality from 20 to three (for the coda type model, Fig. 2) or two (for the vocal clan, Fig. 3, and whale ID, Fig. 4, models). The conspicuous clustering provides qualitative insight into the behavior and efficacy of the trained models, as clustering shows the ability of the NNs to extract features distinguishing different signals. Lastly, we carry out a PCA to better understand the output of the networks and to examine how the networks might be using extracted features to perform the classification tasks (see Supplementary Fig. S5).

GRU and LSTM are similar recurrent network architectures that share the ability to retain memory from previous allocations. However, GRU-based networks are known to be more lightweight and to require fewer parameters, which is advantageous in our situation due to the risk of overfitting. For the coda type and vocal clan classification prediction tasks, we obtain respective training accuracies of 99.6% and 94.1% and testing accuracies of 99.6% and 95.3%. For the whale ID classification problem, we once again evaluate the trained model using the entire training dataset, yielding an accuracy of 99.6%. Noting the similar accuracies as those obtained using the LSTM approach, we investigate the architectures of the various models. The coda type, vocal clan, and whale ID LSTM models respectively contain 932,887, 855,810, and 855,810 total parameters, while the corresponding GRU models involve 735,511, 658,434, and 658,434 parameters. While the classification performance of the GRU-based model is comparable to that of the LSTM-based network, the simpler architecture of the GRU RNN (which is reflected in its relatively fewer trainable parameters) suggests that the GRU approach might provide an improved means to achieve high classification accuracies while avoiding overfitting.

We repeat the LSTM methods using the Eastern Tropical Pacific (ETP) dataset, which contains codas labeled according to type and vocal clan. For the coda type analysis, we obtain an accuracy of 93.6% using 43 distinct coda types. While the entire dataset includes 4,071 codas categorized as non-noise signals, there are a number of relatively rare coda types, which might be challenging to classify correctly. Partitioning the dataset into a disjoint 90/10 training/testing, we train the model on 3,663 codas and obtain 94.2% accuracy, and we test on the remaining 408 unseen codas, yielding an accuracy of 94.4%. For the vocal clan classification task, the model yields an accuracy of 93.1% using 6,044 total codas, 1,511 belonging to each of the four vocal clan classes (two of the vocal clans present in the ETP dataset were discarded due to an insufficient number of recordings present). Repeating the analysis on segmented training (5,741 codas) and testing (303 codas) datasets yields accuracies of 92.5% and 90.4% for training and testing, respectively. These findings demonstrate that the models are able to generalize to unseen datasets with a high degree of robustness.

Lastly, in an effort to address the ability of the NN-based methods to accommodate data from different datasets, we initially pretrain a coda type classification base model using the Dominica dataset and proceed to train and test the network using ETP coda data. Explicitly, using 7,847 Dominica ICI coda vectors with nine or fewer elements, we pretrain a network to perform the usual proxy task, resulting in a mean relative error in final ICI value prediction of 12.4%. After implementing the transfer learning procedure, we prepare segmented training/testing datasets using codas from the ETP (restricting the analysis to coda vectors consisting of fewer than nine elements), and we train the target network to perform the coda type classification. Evaluating the model on training (3,012 codas) and testing (335 codas) datasets yields accuracies of 99.1% and 97.9%, respectively. The results of our study show the feasibility and advantages of using automated ML and NN-based computational approaches to analyze sperm whale acoustic signals.

## Methods

We implement two distinct methods for applying machine learning techniques to sperm whale vocalizations: the first involves a CNN-based approach with spectrogram image inputs to construct an echolocation click detector; and the second uses LSTM and GRU RNNs with time series sequence inputs of coda interclick intervals to classify coda types and to recognize both vocal clans and individual whales. These classification tasks involve supervised learning and transfer learning procedures, during which we train the models using high-quality manually-annotated time-series data.

### Using a CNN-based approach to detect sperm whale echolocation clicks

For the CNN-based method, we construct a sperm whale echolocation click detector designed to label a spectrogram image as “click” or “no click” depending on the network’s classification of the input image. This requires a multi-step procedure involving: (1) processing the raw acoustic data; (2) developing a threshold-based click detector in the time domain; (3) generating an image dataset consisting of appropriately labeled spectrogram images; (4) constructing and training a CNN; (5) testing the trained CNN to assess its ability to generalize to new data; (6) analyzing the robustness of the network and investigating its feature extraction behavior.

We process the raw acoustic data using two publicly available audio datasets: (1) the ‘Best Of’ cuts from the William A. Watkins Collection of Marine Mammal Sound Recordings database from Woods Hole Oceanographic Institution (https://cis.whoi.edu/science/B/whalesounds/index.cfm), and (2) sample sperm whale acoustic recordings from the Centro Interdisciplinare di Bioacustica e Ricerche Ambientali (CIBRA) Cetacean Sound Archive (http://www-3.unipv.it/cibra/res_cesar_uk.html). We initially apply a Butterworth bandpass filter of order five with a low-frequency cutoff f_l_ = 2 kHz and a high-frequency cutoff f_h_ = 20 kHz; this enables us to remove unwanted (low-frequency) noise while preserving the broadband structure of clicks. We subtract the mean and normalize the peak amplitude to process the signal prior to developing our threshold-based click detector algorithm. For the threshold-based detector, we employ a local maximum search algorithm to identify suprathreshold (th = 0.15) peaks separated from nearest neighbors by a temporal distance greater than some canonical minimum ICI value (th_ICI = 0.2 s) determined by inspecting a histogram of detected inter-click time intervals, which allows us to identify clicks while neglecting interferences such as reflections, echoes, and subsequent click pulses. However, the primary motivation for devising this threshold-based method is to generate an appropriately-labeled spectrogram image dataset.

To construct the spectrogram image dataset, we perform the standard spectral analysis and transform to the time-frequency domain. The algorithm automatically labels spectrograms (created using a tapered cosine Tukey window with shape parameter of 0.25, optimized for transient signals), as “click” or “no click” depending on whether or not the threshold-based detector has identified a click as present or absent over the time interval (0.5 s) of the spectrogram. With this approach, we generate a training dataset consisting of 650 images and a testing dataset with 100 spectrograms, ensuring to normalize these datasets with equal numbers of images from each class (“click” and “no click”). Note, however, that a potential source of classification error could be derived from label noise in the training dataset, which should be addressed in a more comprehensive study investigating CNN-based click detectors.

We then proceed to construct the CNN by designing a custom architecture involving three consecutive convolutional and max pooling layers, followed by fully-connected dense layers^56^ (see Supplementary Fig. S6). We include a dropout regularization to prevent potential overfitting. Minimizing the conventional categorical cross entropy objective loss function, we train the network using the annotated training dataset. Performing an exhaustive grid search through the hyperparameter space, we obtain a dictionary of optimal hyperparameters so as to maximize accuracy while still avoiding potential overfitting. For this study, these include filter size 5, batch size 64, a sigmoid activation function, the ‘adam’ optimizer with learning rate 1 × 10^−3^, and 50 training epochs (see^8^ for machine learning fundamentals). Following training, we use the trained model to evaluate the testing dataset as a means to investigate the network’s ability to generalize to unseen data. Finally, after having trained and tested the model, we use standard computational techniques to analyze the feature extraction behavior of the network. Namely, we sequester the high-dimensional hidden features within the network, and we employ a number of dimensionality reduction methods in order to visualize the ability of the network to cluster and classify input data. Firstly, we use PCA to understand how the different features contribute to the variance of the data. Also, we employ PCA to reduce the dimensionality of the hidden feature data from 80 to 20, and then we apply the t-SNE algorithm^57^ to further reduce the dimensionality in order to observe the clustering behavior of the trained model in a two-dimensional plot.

### Using LSTM and GRU RNNs to classify codas, determine vocal clan, and recognize individual whales

Using LSTM RNNs, we construct deep artificial neural networks, which we train to perform a number of classification tasks based on high-quality manually annotated datasets. Data used in this study comes from two long-term field studies on sperm whales: (1) off the island of Dominica in the eastern Caribbean^31^; and (2) across the ETP but with a focus on the Galapagos Islands^58^. The two study sites employed similar methods common in the study of this species to detect, follow, and record sperm whales as well as similar analytical techniques to define coda types, delineate vocal clans, and identify individual whales (details in^29,44,59^). The Dominica dataset contains ~9,000 annotated codas that were collected across 3,834 hours with whales on 406 days over 530 days of effort from 2005–2016. This represents recordings from over 10 different social units who are members of two vocal clans referred to as “EC1” and “EC2”^31,44^. The ETP dataset contains ~17,000 annotated codas collected between 1985–2014 based on recordings of over 89 groups who are members of six vocal clans^29,59^. The annotated datasets contain inter-click interval (the absolute time between clicks in a coda, ICI) vectors classified according to categorical coda type, vocal clan membership, and (in the case of the Dominica dataset) individual whale identity using the methods outlined in the publications listed above. While codas tend to be comprised of 3–40 clicks^27^, we restrict the analysis to codas with at most 10 clicks using the Dominica dataset or at most 12 clicks using the ETP dataset.

Whereas the click detector CNN described above is optimized for image inputs, the LSTM RNN used here accepts time-series sequence inputs. In this case, we use vectors whose elements consist of the ICI values between successive clicks within a given coda. The basic procedure for this time-domain approach to ML-based sperm whale bioacoustic analysis involves (1) pretraining an initial base model to perform a related proxy task and (2) train a model comprising the fixed neural network pretrained on the proxy task and a small trainable neural network to carry out the relevant classification tasks (coda type, vocal clan, and whale identity classification). After training the model, we proceed to test the model and visualize the networks’ activations, primarily by implementing dimensionality reduction algorithms. Initially, we pretrain a custom-built model to perform the proxy task (see Supplementary Fig. S7). In this case, we construct an architecture consisting of two LSTM layers with 256 hidden units followed by a fully connected layer and train the network (by minimizing the root mean square error using the ‘adam’ optimizer with a learning rate 1 × 10^−3^ for 20 epochs) to carry out a supervised time-series forecast. For each coda ICI vector of length *n*, we train the network to predict the *n*th ICI value given the first (*n-1*) values in the sequence. This pretraining procedure enables the network to extract features from the input data, and these features become relevant as we proceed with the transfer learning procedure. To implement transfer learning, we save the deepest (first) layers of the architecture of the pretrained model, and we fix the trained weights as untrainable parameters. After removing the shallowest (last) fully connected layers from the network, we add a supplemental LSTM layer with 256 hidden units (as well as additional fully connected layers) which involve randomly initialized trainable weight parameters. We use both rectified linear unit (ReLU) (ex.^60^) and softmax activation functions (see^8^), and we appropriately adjust the hyperparameters for each task. Using the ground truth labels (coda type, vocal clan, and whale identity) annotated by human experts, we train the model to perform the particular classification task of interest. We seek to minimize the categorical cross entropy loss function in which the network’s predicted label for the coda is compared with the ground truth annotated label. After training the model, we once again test the model on unseen data (so as to address the possibility of overfitting the training dataset), and we also use the standard PCA and t-SNE algorithms to investigate and visualize the feature extraction behavior of the trained model. Finally, we repeat the analysis replacing LSTM layers with GRU layers, and we compare the performances and architectures of the trained models.

## Discussion

The large amounts of high-resolution data collected from increasingly wide regions of the oceans demand novel tools to automate the detection and classification of signals, which can accelerate cetacean bioacoustics research and promote species population assessment and management. Our results show that the sperm whales’ click-based sonar and communication system is well-suited for ML-based techniques. In particular, we establish that CNNs, neural network architectures used for computer vision tasks, can successfully be used to detect sperm whale echolocation clicks from spectrograms. This introduces the prospect of future studies aiming to construct CNN-based models that automatically carry out finer-scale classification tasks (i.e. coda type, vocal clan, whale ID classification) by directly using spectrogram image inputs and avoiding the pre-processing stage of click extraction. This approach to sperm whale classification problems, however, demands large amounts of labeled raw acoustic data, which are not presently available. Instead, we opt to employ RNN-based methods to perform classification tasks on time-series of inter-click intervals. We demonstrate that LSTM and GRU, RNN architectures used for speech recognition and text translation, are able to classify codas into recognizable types and to accurately predict the clan membership and individual identity of the signaler. This is a significant improvement over previous methods used to classify codas as it minimizes the role of the observer in determining any parameters a priori, which was required for coda type statistical clustering techniques involved in prior studies^43,44,59^. Nonetheless, the high coda type classification accuracies we obtain using novel ML-based methods support the previous categorization of sperm whale codas into existing, predefined types. In addition, our results show the feasibility of using “self-supervised” learning on proxy tasks and applying trained neural networks to label unseen coda data according to type, clan, and individual whale, which could drastically expedite the analysis of recorded sperm whale signals.

The datasets in this study collectively contain ~26,000 annotated sperm whale codas and represent the largest available labeled data of sperm whale coda signals. Using these datasets, this study exhibits the potential applications of ML to sperm whale vocalizations by demonstrating the ability of NNs to perform detection and classification tasks with high degrees of accuracy. However, because large volumes of high-quality data are required for training deep neural networks–which aim to achieve state-of-the-art classification accuracy–our analysis is limited to the coda types, clan classes, and individual whales with sufficiently sizable volumes of data to train the networks. In order to improve our methods, even larger datasets would be computationally and methodologically optimal, especially when performing more specialized tasks (e.g. individual whale identification). With that said, such large quantities of high-quality sperm whale data are difficult to obtain (due to the intensive nature of marine fieldwork) as well as to annotate/analyze since such processing tends to require trained human experts. Presently, sperm whale bioacoustics research studies require significant time and oversight by researchers (e.g. to define dialects and extract codas), but the ML-based approaches remain somewhat restricted by the paucity of readily accessible data (including the bulk available data in addition to certain components of the data such as rare coda types). Given the constraints imposed by the limited available data, we show the use of pretraining with proxy tasks as a valid alternative. Our novel techniques achieve high classification accuracies and robustly and effectively generalize to new, unseen data while avoiding overfitting.

This study establishes the numerous advantages that ML-based methods provide for sperm whale bioacoustics research. The techniques presented in this study can also be helpful in the design of next-generation sensors and systems, which should directly intake and analyze sperm whale acoustic data from raw audio recordings. By automating the detection and classification steps, large-scale audio datasets could potentially be processed in near real-time, enabling ML approaches to successfully and efficiently address questions regarding sperm whale vocal behavior and communication (e.g. the cultural distinction between codas produced by different vocal clans) while simultaneously reducing the need for manual oversight.

Given that culture is important for the conservation of wide-ranging species^61^ and that human impacts can greatly erode the cultural diversity within a species^62^, it is critical to have a complete understanding of the cultural boundaries between clans of sperm whales in order to design a framework for the conservation of cultural diversity.The novel ML-based techniques employed in this study enhance the scientific community’s understanding of sperm whale bioacoustics and provide an efficient means for analyzing large datasets, which can facilitate the development of improved conservation and management strategies designed to protect global sperm whale (and other cetacean) populations.

## Supplementary information

Fig S1-S7 and Table S1 Supplementary Information

S6

S7

## Data Availability

The Dominica coda dataset used to train and evaluate the neural networks is available as Supplementary File S6 and the ETP coda dataset used to train and evaluate the neural networks is available as Supplementary File S7. The custom-written algorithms (Python 3.0) used in this study are available at: https://github.com/dgruber212/Sperm_Whale_Machine_Learning. All data generated or analyzed during this study are included in this published article and its Supplementary Information files. The machine learning code is available on Github.
