# Publisher Correction: Deep Machine Learning Techniques for the Detection and Classification of Sperm Whale Bioacoustics

**DOI:** 10.1038/s41598-019-53408-7

**Published:** 2019-12-06

**Authors:** Peter C. Bermant, Michael M. Bronstein, Robert J. Wood, Shane Gero, David F. Gruber

**Affiliations:** 1 Radcliffe Institute for Advanced Study, Harvard University, Cambridge, MA USA; 2 Department of Computing, Imperial College, London, UK; 3 Wyss Institute for Biologically Inspired Engineering, Harvard University, Cambridge, MA USA; 4 Harvard John A. Paulson School of Engineering and Applied Sciences, Harvard University, Cambridge, MA USA; 5 Department of Zoophysiology, Institute for Bioscience, Aarhus University, C.F., Møllers Allé 3, Aarhus, 8000 Denmark; 6 Department of Natural Sciences, Baruch College and The Graduate Center, PhD Program in Biology, City University of New York, New York, NY USA; 7 Twitter, 20 Air Street, London, W1B 5DL United Kingdom

Correction to: *Scientific Reports* 10.1038/s41598-019-48909-4, published online 29 August 2019

In the original version of this Article, the ORCID ID for Shane Gero was omitted.

In addition, Supplementary Information file S6 was omitted from the original version of this Article. These errors have been corrected in the HTML and PDF versions of the Article.

