# Supplementary material for: Deep Machine Learning Techniques for the Detection and Classification of Sperm Whale Bioacoustics
Source: Sci Rep. 2019 Aug 29;9:12588. doi: 10.1038/s41598-019-48909-4 (PMC6715799; doi:10.1038/s41598-019-48909-4)
Supplement: Supplementary file 1 — Fig S1-S7 and Table S1 Supplementary Information [file 41598_2019_48909_MOESM1_ESM.pdf]

Supplementary Information

Deep Machine Learning Techniques for the Detection and Classification of Sperm Whale Bioacoustics

Peter C. Bermant[1], Michael M. Bronstein[1,2], Robert J. Wood[3,4], Shane Gero[5#], David F. Gruber[1,6#*]

[1]Radcliffe Institute for Advanced Study, Harvard University, Cambridge, MA, USA
[2]Department of Computing, Imperial College, London, UK
[3]Wyss Institute for Biologically Inspired Engineering, Harvard University, Cambridge, MA, USA
[4]Harvard John A. Paulson School of Engineering and Applied Sciences, Harvard University, Cambridge, MA, USA
[5]Department of Zoophysiology, Institute for Bioscience, Aarhus University, C.F. Møllers Allé 3, Aarhus 8000, Denmark
[6]Department of Natural Sciences, Baruch College and The Graduate Center, PhD Program in Biology, City University of New York, New York, NY, USA

[#]contributed equally as senior authors
[*]Correspondence to: David.Gruber@baruch.cuny.edu

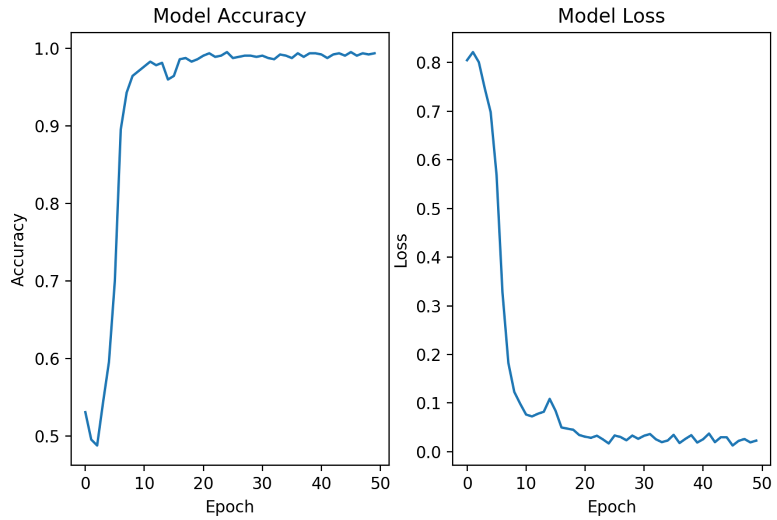

Fig. S1: Training the CNN-based echolocation click detector involves adjusting model weights to optimize the objective loss function, which, in this case, is the categorical cross entropy function. As training progresses, the accuracy of signal classification exhibits an overall increasing trend.

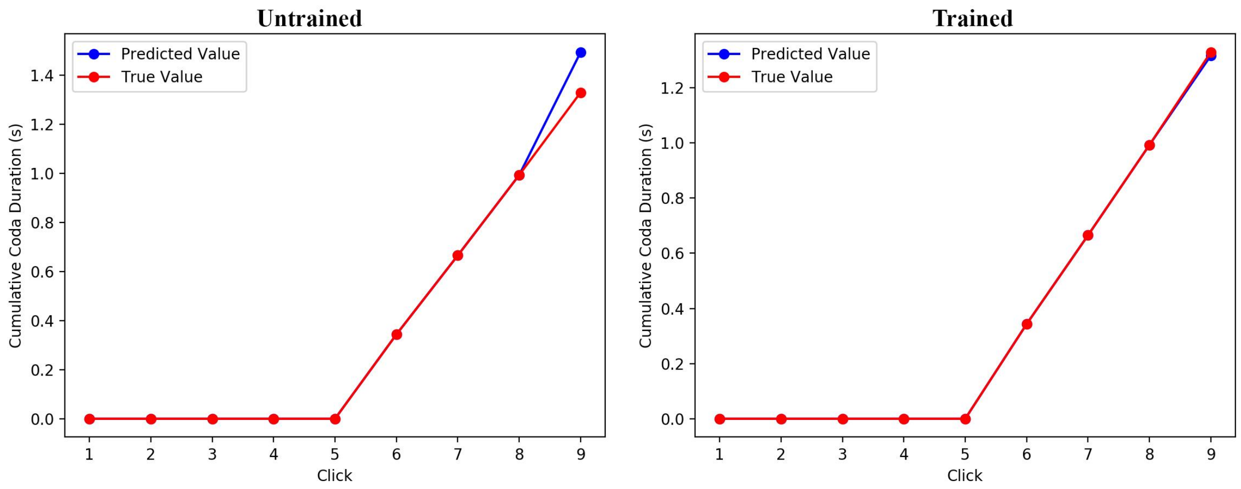

Fig. S2: The pretrained network predicts the temporal position of the final *n*th click in a *n*-click coda, given as inputs the first *n-1* clicks of the coda. The absolute relative error can be reduced from ~300% to ~12.5% following training.

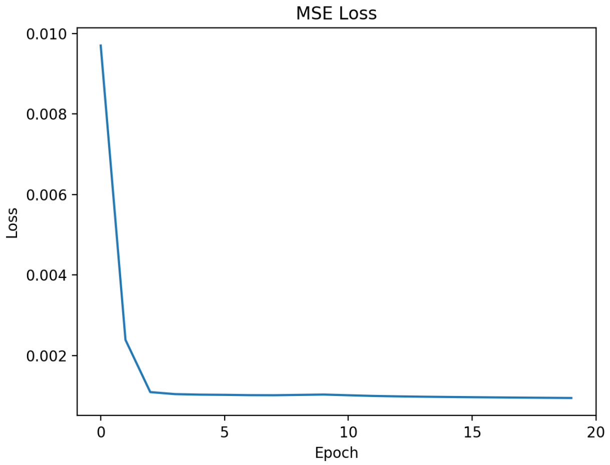

Fig. S3: Pretraining the proxy task base model involves minimizing the mean squared error (MSE) objective loss function, which is calculated by comparing the predicted final inter-click interval (ICI) value with the actual value. The model involves 789,761 total parameters, of which all are trainable.

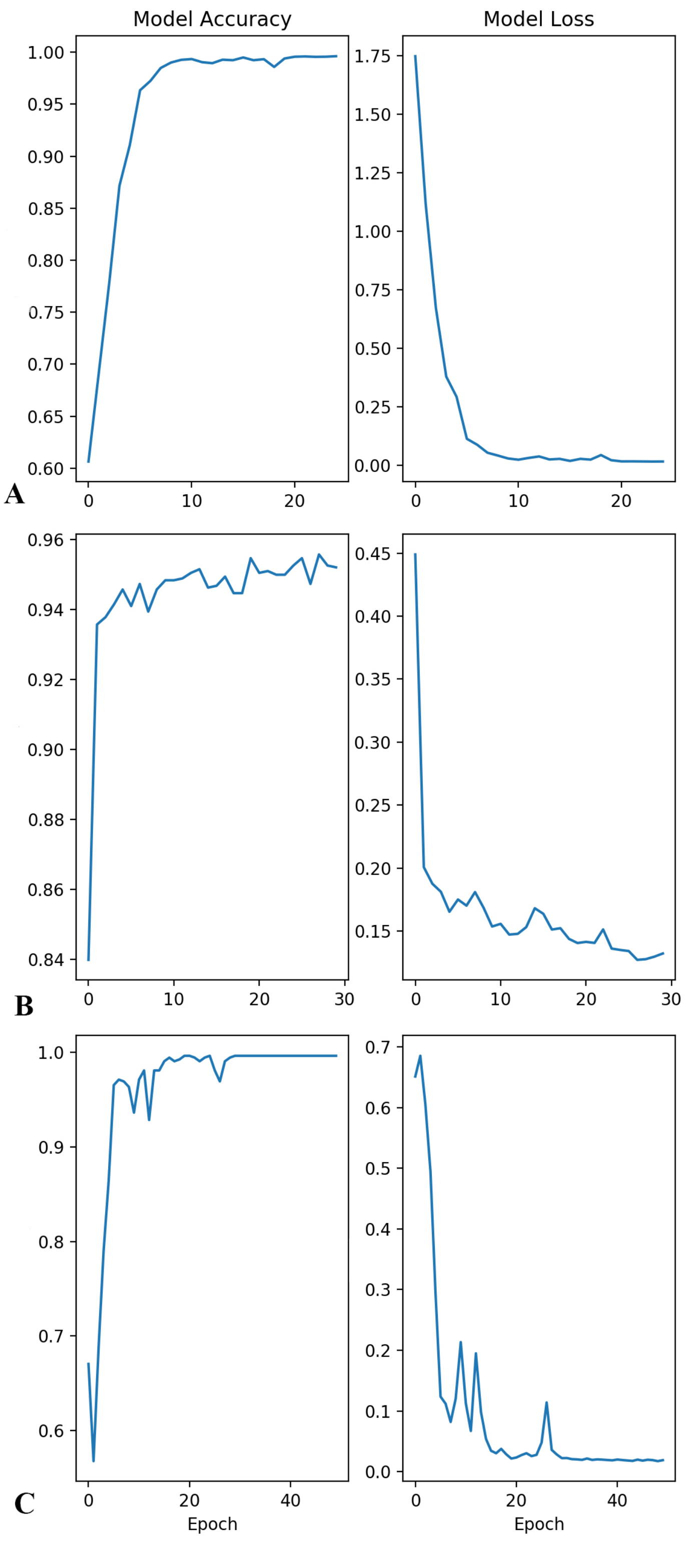

Fig. S4: A) Training the coda type classification model involves minimizing the categorical cross entropy objective loss function. The transfer learning procedure yields a pretrained model with 264,192 non-trainable parameters, and additional shallow layers involve 668,695 trainable

parameters that are adjusted during model fitting. B) Training the vocal clan classification model. The transfer learning procedure in combination with the construction of the clan class model results in a network with 855,810 total parameters, of which 591,618 are trainable and 264,192 are non-trainable. C) Training the whale ID classification model. The transfer learning procedure used to construct the whale ID model yields a deep network with 855,810 total parameters, of which 591,618 are trainable.

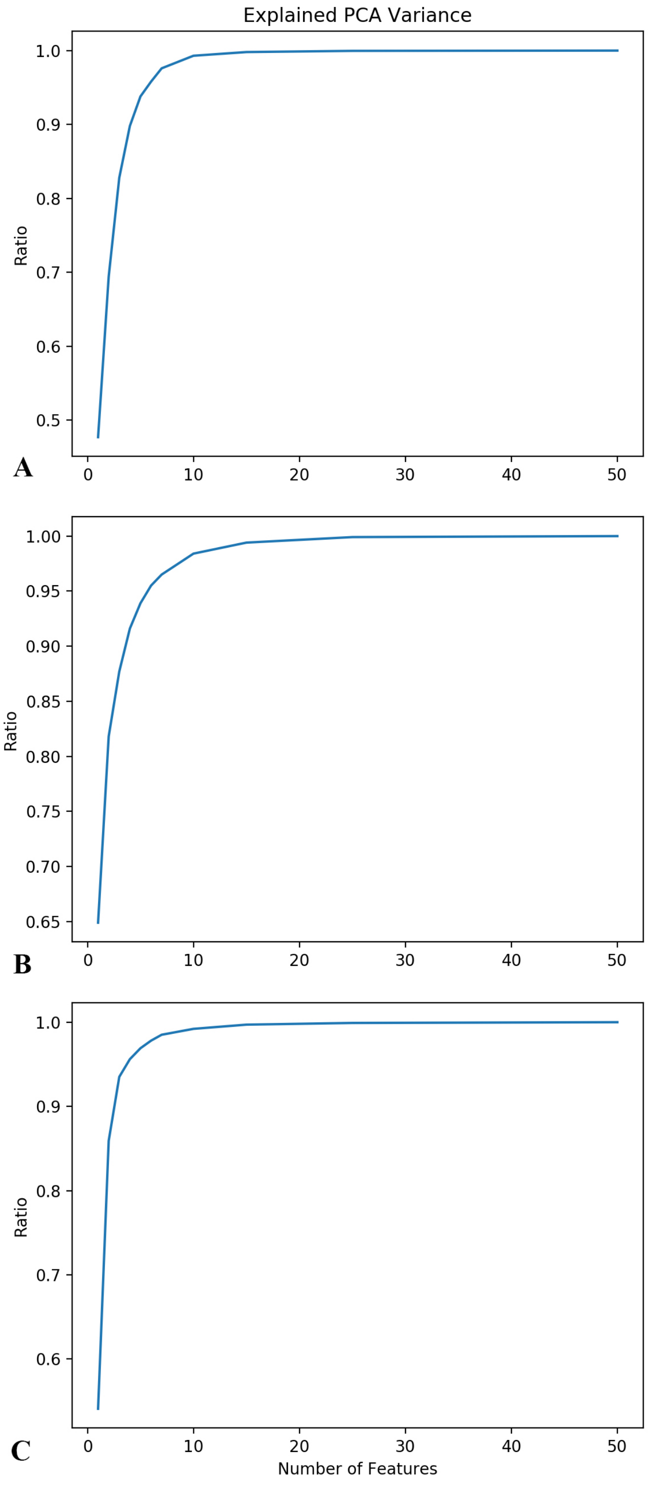

Fig. S5: A) Explained PCA variance for the coda type model. The first component contributes to 47.7% of the explained variance, while including 10 components results in 99.3% of the explained variance. B) Explained PCA variance for the clan class model. The first component

contributes to 64.9% of the explained variance, while including 15 components results in 99.4% of the explained variance. C) Explained PCA variance for the whale ID model. The first component contributes to 54.1% of the explained variance, while including 10 components results in 99.2% of the explained variance.

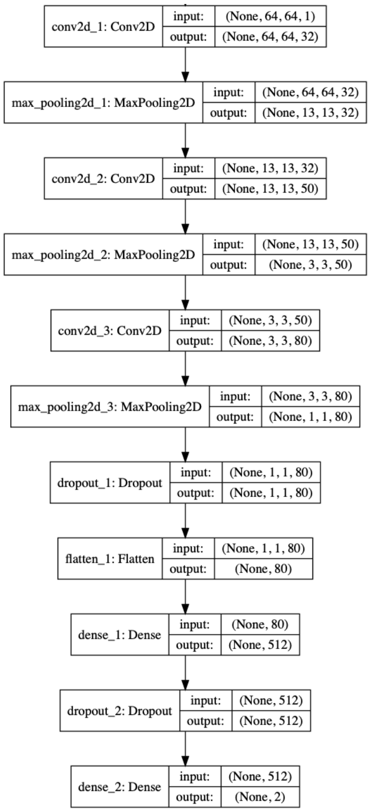

Fig. S6: Architecture of the CNN-based echolocation click detector

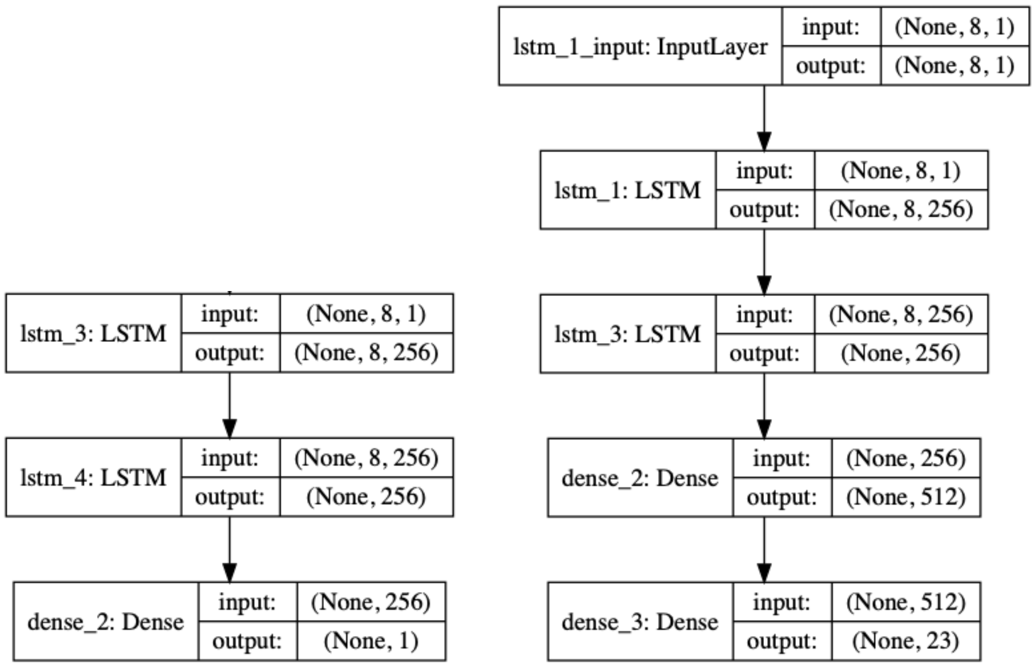

Fig. S7: Architecture of A) the pretrained base model and B) the target model.

Supplementary Data

File S6: Dominica coda dataset used to train and evaluate the neural networks.

File S7: ETP coda dataset used to train and evaluate the neural networks.

Supplementary Table 1. List of acronyms used in study.

Convolutional Neural Network	CNN
Deep Learning	DL
Eastern Tropical Pacific	ETP
Gated Recurrent Unit	GRU
Gaussian Mixture Model	GMM
Hidden Markov Models	HMM
Inter-Click Interval	ICI
Long Short-Term Memory	LSTM
Machine Learning	ML
Multi-Layer Perceptron	MLP
Neural Network	NN
Principal Component Analysis	PCA
Recurrent Neural Network	RNN
Support Vector Machine	SVM
t-Distributed Stochastic Neighbor Embedding	t-SNE
